# Exsection of Inferior Dental Nerve, for Relief of Paroxysms in Lower Jaw

**Published:** 1886-02

**Authors:** L. P. Dottere

**Affiliations:** South Carolina.


ARTICLE V.

### EXSECTION OF INFERIOR DENTAL NERVE, FOR RELIEF OF PAROXYSMS IN LOWER JAW.

BY L. P. DOTTERE, D. D. S., OF SOUTH CAROLINA.

(Read before the Charleston Dental Association.)

HISTORY.—Patient, sixty-four, having lost all her teeth in trying to gain relief; but since removal of the last, which was about eight years ago, pain has been augmented, though intermittent; since she would have a few months' relief, and then as many in bed, suffering intensely every few minutes with paroxysmal pains, located in the jaw between the position of second bicuspid and median line. Her lower lip on affected side was also troubled, and the act of swallowing or pressure upon the jaw would bring on a paroxysm. Nourishment had to be of a lukewarm temperature, and any thermal change would aggravate the pain. Her attending physician called me in consultation, and after a thorough examination, diagnosed the inferior dental nerve as being stretched, or having a bulbous formation.

Knowing the patient to be averse to an operation, advised a hard rubber capping, to extend all around the jaw, and the wearing under this of a layer of bibulous paper saturated with cocaine.

This relieved her somewhat, and assisted in swallowing but did not stop paroxysms, which continued nearly as intense, especially early in the morning.

After testing this treatment, advised the operation which is the subject of this paper.

The patient being chloroformed, an incision was made from the position of the second molar to bicuspid along the lingual border of the bone, then another transversely towards the cheek: The integment was then pressed back, exposing the upper surface of the bone.

Through this was made two parallel cuts with a circular saw, guarded by a shield, and worked by the dental engine. Next, the ends of these cuts were joined by a fissure drill, and the block of bone removed, exposing the canal. The nerve was then raised with a hooked excavator, and cut in two places, taking away about a quarter of an inch.

The edges of the bone were then rounded with a large bur, parts well syringed with tepid water, and the flesh wound stitched ; but before the patient became conscious, we injected into the canal about twenty drops of cocaine, to relieve after pain.

Next day patient complained of a throbbing and knitting sensation, but wound showed no signs of sloughing. In one week the stitches were removed, and in two weeks wound had healed by resolution.

Dr. C. B. Lannear ably assisted in the operation, which was performed five weeks ago, and since, there has not been a paroxysm ; though she still feels at intervals a gnawing sensation, which no doubt is caused by nature in bridging over the canal.—*Southern Dental Journal.*

